# Characterization of Active Packaging Films Made from Poly(Lactic Acid)/Poly(Trimethylene Carbonate) Incorporated with Oregano Essential Oil

**DOI:** 10.3390/molecules21060695

**Published:** 2016-05-27

**Authors:** Dong Liu, Hongli Li, Lin Jiang, Yongming Chuan, Minglong Yuan, Haiyun Chen

**Affiliations:** Engineering Research Center of Biopolymer Functional Materials of Yunnan, Yunnan Minzu University, Kunming 650500, Yunnan, China; liudong5988@126.com (D.L.); honglili_1982@163.com (H.L.); jianglin_1981@163.com (L.J.); chuan5211017@126.com (Y.C.)

**Keywords:** poly(lactic acid), poly(trimethylene carbonate), oregano essential oil, antioxidant, antimicrobial, biodegradable film, food packaging

## Abstract

Antimicromial and antioxidant bioactive films based on poly(lactic acid)/poly(trimenthylene carbonate) films incorporated with different concentrations of oregano essential oil (OEO) were prepared by solvent casting. The antimicrobial, antioxidant, physical, thermal, microstructural, and mechanical properties of the resulting films were examined. Scanning electron microscopy analysis revealed that the cross-section of films became rougher when OEO was incorporated into PLA/PTMC blends. Differential scanning calorimetry analysis indicated that crystallinity of PLA phase decreased by the addition of OEO, but this did not affect the thermal stability of the films. Water vapor permeability of films slightly increased with increasing concentration of OEO. However, active PLA/PTMC/OEO composite films showed adequate barrier properties for food packaging application. The antimicrobial and antioxidant capacities were significantly improved with the incorporation of OEO (*p* < 0.05). The results demonstrated that an optimal balance between the mechanical, barrier, thermal, antioxidant, and antimicrobial properties of the films was achieved by the incorporation of 9 wt % OEO into PLA/PTMC blends.

## 1. Introduction

Active packaging materials with antioxidant or antimicrobial properties have gained considerable attention, since they are one of the most promising alternatives to traditional packaging [1]. Meanwhile, biodegradable active packaging can provide a solution to the environmental problem of solid waste resulting from the use of non-biodegradable petrochemical-based plastic packaging materials [2,3]. Biodegradable plastics such as poly(lactic acid) (PLA), poly(ε-caprolactone) (PCL), poly(ethylene glycol) (PEG) and polypropylene glycol (PPG) are noteworthy materials is this respect [4]. Among them, PLA, a biodegradable polymer which can be generated from renewable resources such as corn starch or sugarcane has recently attracted growing attention [5,6,7,8]. Because of its relatively low cost, processibility and biocompatibility, it is an interesting candidate for producing food packaging materials [9].

However, some characteristic properties of pure PLA such as weak thermal stability, low gas barrier properties, and low ductility and toughness are inadequate for food packaging applications [10]. Poly(trimethylene carbonate) (PTMC) is an amorphous biodegradable polymer, which has a low glass transition temperature (*T_g_*) between −14 and −25 °C. High-molecular-weight PTMC displays elastic properties at ambient temperature [11,12,13]. Qin *et al.* found that 70/30 PLA/PTMC blends enhanced the elongation at break of PLA-based films [14]. The addition of the PTMC thus makes the composite films become softer and more elastic.

Current innovation in active packaging has focused on the development of active packaging systems based on materials which can include a variety of additives with the objective of extending foodstuff shelf-life [15]. The interesting demand for natural additives has resulted in studies based on natural active compounds such as plant extracts or essential oils, which are categorized as Generally Recognized as Safe (GRAS) by the U.S. Food and Drug Administration as well as the current European Legislation for materials intended to be in contact with food (EU N10/2011 Regulation) [16]. The films incorporating essential oils could serve as a protective barrier for contaminating microorganisms on the surface of food and reduce lipid oxidantion for the subsequent improvement of quality and safety of coated products. Essential oils are regarded as natural alternatives of chemical preservatives and meet the demands of consumers for mildly processed or natural products in the use of food packaging.

The use of oregano essential oil is a commendable alternative to conventional antioxidant and antimicrobial agents. The antioxidant and antimicrobial properties of oregano essential oil, which result from its high content of phenolic compounds, have been extensively reported. [17,18]. It has been used as a raw material of drugs and health care products worldwide.

In this study, PLA/PTMC/OEO composite films, containing 0, 3, 6, 9 and 12 wt % oregano essential oil, and identified as PLA/PTMC/On, where *n* = %OEO, have been prepared by a solvent casting method. The effect of oregano essential oil content on the physical, thermal, mechanical, antioxidant and antimicrobial properties was explored. The results point out that the incorporation of oregano essential oil as a natural antimicrobial agent has potential for use in composite films for bioactive packaging materials.

## 2. Results and Discussion

### 2.1. SEM Analysis

**Figure 1:**
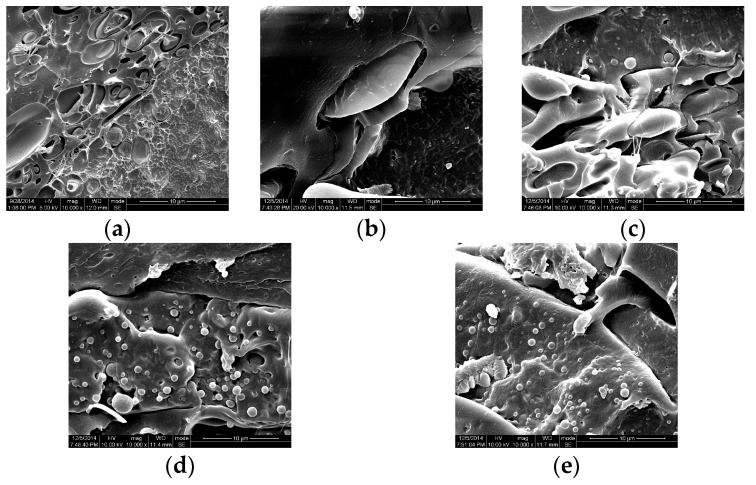
SEM micrographs of the fracture surfaces morphology of (**a**) PLA/PTMC; (**b**) PLA/PTMC/O3; (**c**) PLA/PTMC/O6; (**d**) PLA/PTMC/O9; and (**e**) PLA/PTMC/O12 (magnification: 10,000×).

SEM can provide a better understanding of the relationships between the water vapor transmission, mechanical properties and the film’s structural characteristics. The effect of OEO dispersion into the polymer matrix can be observed in Figure 1b–e.

Figure 1a shows that the fracture surface of PLA/PTMC blend was irregular. This might be because that the PLA and PTMC were partially miscible and PTMC promoted the separation of the aggregated PLA particles during blending [19,20]. However, the fracture surface of films with OEO showed a heterogeneous structure in which oil droplets were entrapped in the continuous polymeric network. The images of PLA/PTMC/O3, PLA/PTMC/O6, PLA/PTMC/O9 and PLA/PTMC/O12 (Figure 1b–e) showed that the addition of OEO to PLA/PTMC led to changes in the structure of films. With the addition of OEO, the cross-section of film became rougher, compared with the PLA/PTMC composite films. This might be because a certain amount of the OEO in the composite partialtly evaporated from the polymer matrix during processing. Liakos *et al.* [21] observed phase blending and interaction as well as the formation of essential oil domains in NaAlg/glycerol/lgepal/lemongrass oil intermediate EO concentration film by atomic force microscopy (AFM), and they found the essential oils were stably encapsulated within the films. For antimicrobial and antioxidant active packaging, the resulting films might facilitate the release of the antimicrobial and antioxidant agent.

### 2.2. DSC Analysis

**Figure 2:**
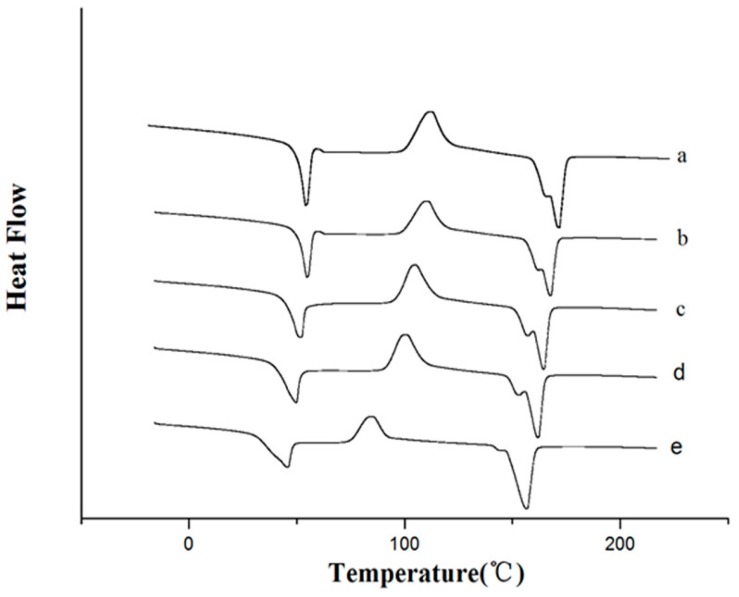
Differential scanning calorimetry curves of poly (lactic acid)/poly(trimethylene carbonate)/oregano essential oil composite films (**a**) PLA/PTMC; (**b**) PLA/PTMC/O3; (**c**) PLA/PTMC/O6; (**d**) PLA/PTMC/O9 and (**e**) PLA/PTMC/O12.

In this study, DSC measurements were carried out to investigate the thermal transitions of the films. The glass transition temperature (*T_g_*), peak crystallization temperature (*T_c_*), and melting temperatures (*T_m_*) of the tested materials are summarized in Table 1. *T_g_* was determined from the second heating scan, once the thermal history was erased in the first heating scan. The DSC scan curves of PLA/PTMC and PLA/PTMC/OEO are shown in Figure 2. The *T_g_* of PTMC was reported to be −24 °C [22], but the pure PLA exhibited a glass transition around 57 °C. The difference in the *T_g_* between PTMC and PLA was about 70 °C. In this study, both *T_g_s* was between the *T_g_s* of the two component polymers. With the incorporation of OEO, the *T_g_* value of PLA/PTMC composite films decreased from 46.7 to 45.6 °C. In the one hand, this indicated that the PLA/PTMC/OEO composites were partially miscible, and on the other hand, it confirmed that OEO exerted a plasticizer effect. The second low temperature melting peak of PLA in the composites also confirmed the PTMC acted as a plasticizer of PLA [22].

The *T_c_* value and melting temperature increased first and then decreased with the increasing amount of OEO. The *T_c_* of PLA/PTMC/OEO12 decreased 20.1% compared to the values obtained for the unfilled PLA/PTMC blends. As observed, the melting enthalpy of PLA/PTMC was higher than that of the blends incorporated with OEO, which could indicate a higher crystallinity of PLA/PTMC without OEO.

The value of *X_c_* is also shown in Table 1. The *X_c_* of PLA/PTMC was higher than that of PLA/PTMC/OEO composites, so it could be concluded that the crystallinity of the composites decreased with the addition of OEO. The plasticization effect of OEO was also observed in the tensile tests, as discussed in the mechanical properties section. This might be because of the interaction between the polymer matrix and additive molecules in the polymer macromolecular network [9].

### 2.3. Thermogravimetric Analysis

**Figure 3:**
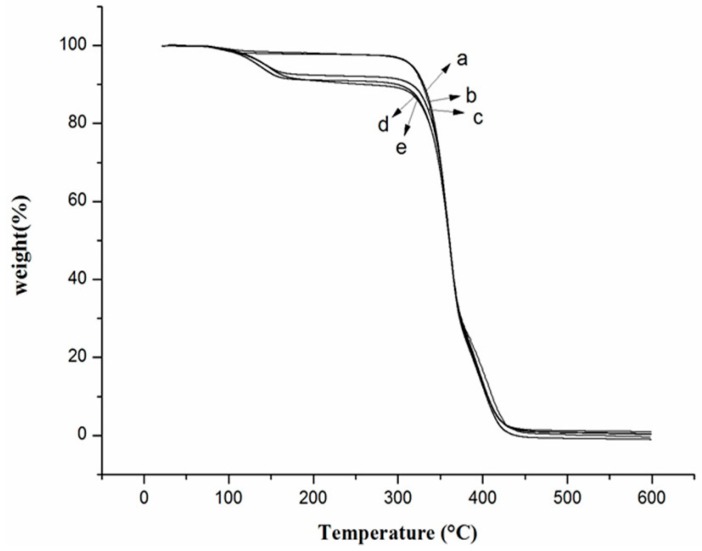
Thermogravimetric analysis curves of poly (lactic acid)/poly (trimethylene carbonate)/oregano essential oil composite films (**a**) PLA/PTMC; (**b**) PLA/PTMC/O3; (**c**) PLA/PTMC/O6; (**d**) PLA/PTMC/O9 and (**e**) PLA/PTMC/O12.

The thermal degradation of PLA/PTMC/OEO samples is shown in Figure 3. A slight decrease in weight for PLA/PTMC/OEO below 180 °C was evident, which was due to the evaporation of the OEO incorporated in the composites. The weight loss from the first step of the PLA/PTMC was 96.3 wt %, when 12 wt % OEO was incorporated into the PLA/PTMC matrix, and the weight loss from the first step of the sample was 91.52 wt %. The *T_onset_* of PLA/PTMC is 293 °C, and there were slightly differences in *T_onset_* values between PLA/PTMC and PLA/PTMC/OEO composites. However, the TGA thermograms indicated that all composites were essentially thermally stable below 180 °C. Wu *et al*. [23] also reported that the TGA of PLA/PCL/thymol composites showed no apparent differences. In practice, when consumers use PLA-based materials for food packaging, they are always stored at room temperature or even below, so the thermal stability of PLA/PTMC/OEO composites is not influenced.

### 2.4. Color

**Figure 4:**
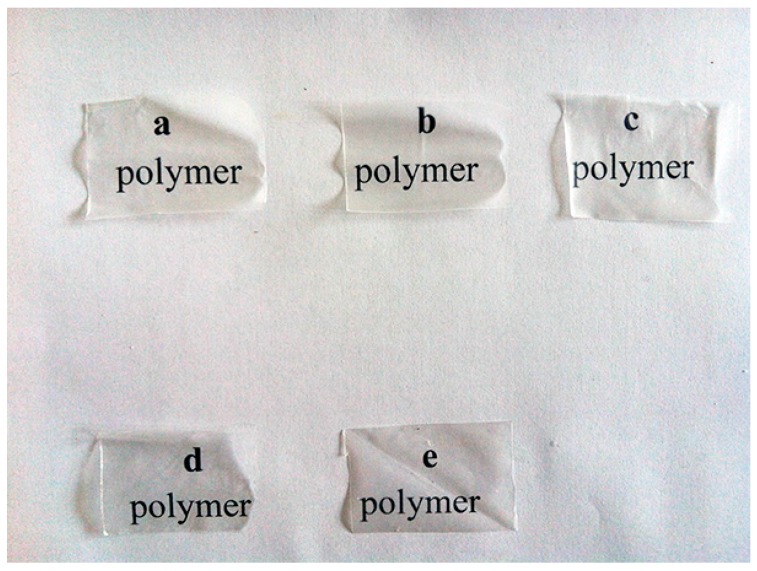
Color of (**a**) PLA/PTMC; (**b**) PLA/PTMC/O3; (**c**) PLA/PTMC/O6; (**d**) PLA/PTMC/O9 and (**e**) PLA/PTMC/O12.

The color and opacity of PLA/PTMC/OEO composite films are shown in Table 2. Generally, there was a significant (*p* < 0.05) difference in L* (lightness) between each sample. The a* (redness) value showed no significant (*p* > 0.05) increase as the OEO increased. The transparency of the PLA/PTMC/OEO composite films decreased significantly (*p* < 0.05) compared to the PLA/PTMC film. This might be attributed to the clear yellowish color of OEO. The decreased transparency of composite films as a result of the addition of natural antimicrobial or antioxidant agents has also been reported for PLA-based films [10]. The visual appearance of PLA/PTMC and PLA/PTMC/OEO composite films is shown in Figure 4. Statistically significant differences were found among samples with OEO, however, these differences were not perceptible to the human eye (Figure 4). This result showed high transparency for PLA/PTMC/OEO composite films, and this aspect of packaging could influence the decisions of most consumers.

### 2.5. Antioxidant Activity and Total Phenolic Content

**Figure 5:**
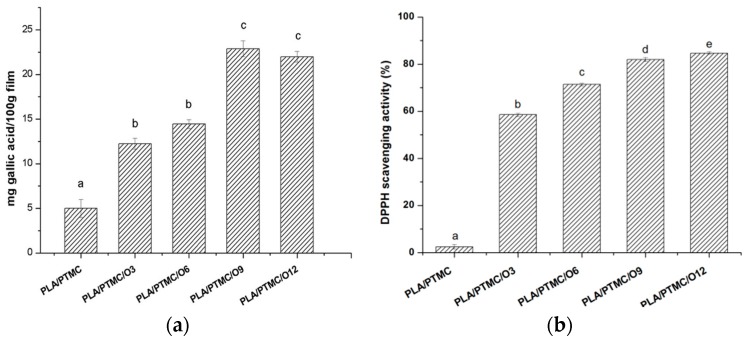
(**a**) Total phenolic content of PLA/PTMC, PLA/PTMC/O3, PLA/PTMC/O6, PLA/PTMC/O9 and PLA/PTMC/O12; (**b**) DPPH scavenging activity of PLA/PTMC, PLA/PTMC/O3, PLA/PTMC/O6, PLA/PTMC/O9 and PLA/PTMC/O12. Values followed by different letters (a–e) were significantly different (*p* < 0.05).

The total phenolic (TP) content of PLA/PTMC/OEO composites is shown in Figure 5a. The TP content of PLA/PTMC film was 4.99 mg GAE/100 g PLA/PTMC/OEO composite films. TP content in the composite films significantly increased (*p* < 0.05) with the increasing oregano essential oil content. The TP contents of PLA/PTMC/O3, PLA/PTMC/O6, PLA/PTMC/O9 and PLA/PTMC/O12 were 12.25, 14.45, 22.9 and 22.0 mg/100 g composite film, respectively. The antioxidant activities of composite films were also investigated using the DPPH radical scavenging assay, since it is necessary to combine more than one method in order to determine *in vitro* the antioxidant capacity of foodstuffs [24]. This has become a general test method for the measurement of the free radical scavenging ability of films added with plant essential oils. Figure 5b shows the antioxidant activity of composite films. The PLA/PTMC blend had a slight radical scavenging activity. The results showed that the DPPH scavenging activity of the films increased significantly (*p* < 0.05) as oregano essential oil concentration increased, the antioxidant values varied from 58.57% to 84.7%. The highest antioxidant activity was obtained for the films containing 12%, which has a little higher than the film containing 9%. Moradi *et al.* found that the degree of antioxidant power of edible films is generally proportional to the amount of added antioxidant additives [25]. However, these results were expressed in 2 mg of film, showing the PLA/PTMC/OEO composite films presented a powerful scavenging activity, which was higher than the value of 61.03% in previous reports [18]. This result revealed strong scavenging activity resulting from the addition of antioxidants to biofilms. Gómez-Estaca also pointed that the degree of antioxidant power of biodegradable film is generally proportional to the amount of added antioxidant additives [26].

### 2.6. Antimicrobial Results

There have been a number of studies on the antimicrobial activity of films incorporated with essential oil such as *Mentha pulegium* essential oil, cinnamon essential oil or other essential oils [27,28], but there have been a few studies on active films incorporating oregano essential oil. Salmieri *et al.* just studied the effect of oregano essential oil against *Listeria monocytogenes* [29]. The antimicrobial activities of the composite films were tested against the Gram positive bacteria *E. coli* and *L. monocytogenes* as *E. coli* and *L. monocytogenes* represent typical spoilage organism groups commonly occurring in food products [23].

**Figure 6:**
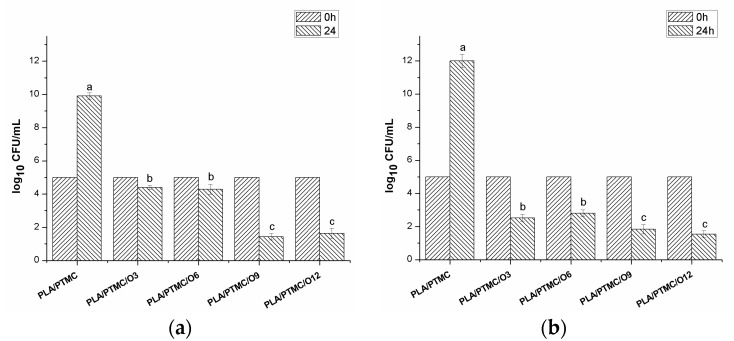
Antimicrobial activity of poly(lactic acid)/poly (trimenthylene carbonate)/oregano essential oil composite films (**a**) *Listeria monocytogenes*; (**b**) *Escherichia coli*. Values followed by different letters (a–c) were significantly different (*p* < 0.05).

Figure 6a,b show the PLA/PCL blend without oregano did not inhibit bacterial growth, whereas films containing 9 wt % OEO were significantly effective against *E. coli* and *L. monocytogenes* compared with other samples at 24 h. As could be seen, after 24 h of incubation, the PLA/PTMC/O9 had already reduced *E. coli* from 5.0 to 1.4 logs and *L. monocytogenes* from 5.0 to 1.5 logs. This result strongly confirms that the films containing 9 wt % or higher OEO demonstrate a strong antimicrobial capacity against *E. coli* and *L. monocytogenes*. This might be because the gradual release of the molecules over time allowed their continuous availability and partitioning into the cell membranes [9]. The antimicrobial effect of OEOs can be attributed to the concentration and proportion of phenolic compounds, especially carvacrol and thymol, which are the most active constituents of oregano essential oil, with a wide spectrum of demonstrated antimicrobial properties [30,31,32]. The screening of antimicrobial activity of OEOs has shown that they are some of the most effective antibacterial agents [33,34,35,36,37,38]. In this study, we demonstrated that the OEO incorporated in PLA/PTMC composites has a crucial effect on antimicrobial activity.

### 2.7. Mechanical Properties

Sufficient flexibility to avoid breaking during food packaging is very important for polymer films. The effect of OEO concentration on the mechanical properties was evaluated by the TS, E and tensile modulus of the films (Table 3).

The neat PLA was found to be brittle. Conversely, PTMC polymer had good ductility. The brittleness of PLA was obviously improved by blending it with PTMC. When OEO was incorporated into the PLA/PTMC polymer matrix, the TS of PLA/PTMC/OEO composites did not significantly (*p* > 0.05) change. When the material was applied to food packaging, it was not easy to crack and could efficiently prevent foods from deterioration.

The E values of the neat PLA/PTMC blends were found to be 105.51%, however, the E values of PLA/PTMC composites incorporated with OEO were significantly (*p* < 0.05) increased by 68.7% and 71.9% with the addition of 3 wt % and 6 wt % OEO, respectively. Upon the addition of 9 wt % OEO, the E value increased by 77.0%. This might be because that the high OEO content had a plasticizer effect. This had also been noted in the DSC results.

These differences on the mechanical behavior of films with EOs were also observed by Kavoosi *et al.*, who found that gelatin films incorporated with *Zataria multiflora* essential oil had a lower tensile strength but higher elongation at break compared with film without essential oil [39]. The addition of essential oil resulted in a lowered interaction between PLA molecules and hindered polymer chain-to-chain interactions. As a result, this would cause a significant decrease in the tensile strength of the films [40].

### 2.8. WVP

**Figure 7:**
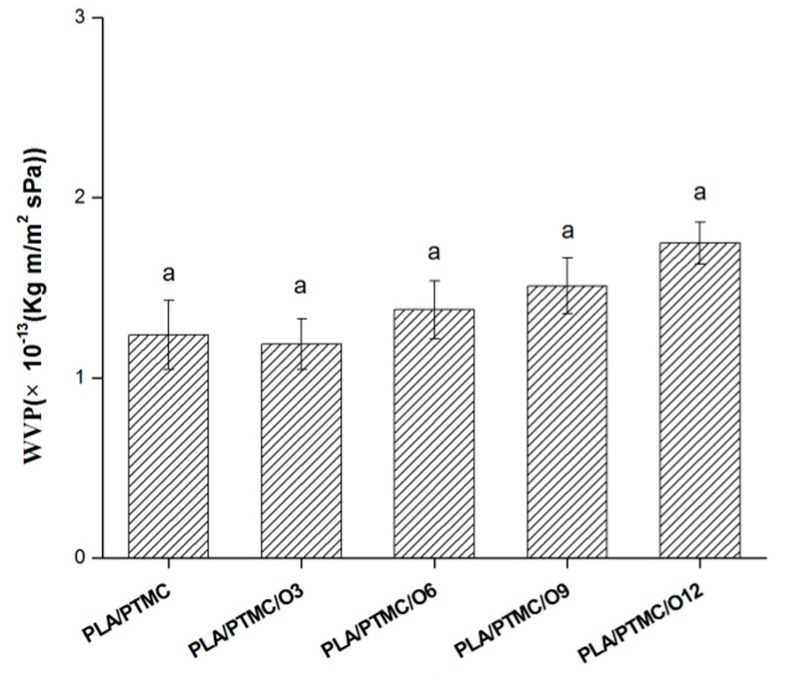
Water vapor permeability (WVP) of PLA/PTMC, PLA/PTMC/O3, PLA/PTMC/O6, PLA/PTMC/O9 and PLA/PTMC/O12 film. Values followed by same letter (a) was not significantly different (*p* > 0.05).

Water vapor permeability (WVP) is one of the most important properties in food packaging because of the noticeable role of water in deteriorative reactions and microbial growth [41]. The effect of the OEO amount on the WVP of PLA/PCL blends is shown in Figure 7. WVP increased from 1.24 × 10^−13^ to 1.74 × 10^−13^ (kg·m/m^2^·s·Pa) as the OEO content was increased from 0 to 12 wt %. It was reported that incorporation of limonene as plasticizer led to a decrease in barrier properties [42]. Similarly, Wu *et al.* reported that the WVPs of PLA/PTMC composite films were increased by the addition of thymol as antimicrobial agent. This might be attributed to the fact that the addition of OEO increased the average pore size of the films [23]. Kavoosi *et al.* also reported that the incorporation of thymol, especially at higher content, caused a significant increase (*p* < 0.05) in the WVP of gelatin films [39]. As previously reported, a slight decrease in RH within the packing due to the high water vapor permeability of the film would be beneficial for post-harvest vegetable and fruit quality [43]. No condensation was observed when using materials with higher water vapor permeability, so the active PLA/PTMC/OEO composite films showed adequate barrier properties and could be acceptable for food packaging.

## 3. Materials and Methods

### 3.1. Materials

Commercial poly(lactic acid) (PLA, M_w_ = 280kDa, M_w_/M_n_ = 1.98) from Natureworks LLC (Blair, NE, USA) was used as the biodegradable matrix. Poly(trimethylene carbonate) (PTMC, M_w_ = 100 kDa, M_w_/M_n_ = 1.70) was synthesized in our laboratory. According to previous reports, [13,14] using trimethylene carbonate as monomer and 1.5‰ stannous octoate as catalyst, polytrimethylene carbonate (PTMC) was synthesized by ring-opening polymerization under the 130 °C for 4 h. Oregano essential oil was supplied by dōTERRA Co., Ltd. (New York, NY, USA). Chloroform was obtained from Chengdu Kelong Chemical Co., Ltd. (Sichuan, China). Folin-Ciocalteu reagent, sodium carbonate, gallic acid standard and 2,2-diphenyl-1-picrylhydrazyl (DPPH) were purchased from Sigma Chemical Co. (St. Louis, MO, USA). All other reagents used in this study were of analytical grade.

### 3.2. Preparation of Films

The materials were pre-treated by drying in a vacuum oven at 50 °C for 24 h to eliminate possible absorbed water on the surface of the particles. PLA/PTMC (70/30, 2 g) was dissolved in chloroform (50 mL). Then, 0, 3, 6, 9 and 12 wt % oregano essential oil were added in PLA/PTMC chloroform solution and stirred until all the oregano essential oils were dissolved. Then the solutions were placed on a polytetrafluoroethylene (PTFE) plate, and the solvent used was evaporated at 60 °C for 24 h to volatilize the residual solvent and then the films were cut into 100 mm × 25.4 mm pieces for the investigation of film properties. PLA/PTMC incorporated with OEO at 0, 3, 6, 9 and 12 wt % loading were named as PLA/PTMC, PLA/PTMC/O3, PLA/PTMC/O6, PLA/PTMC/O9 and PLA/PTMC/O12, respectively.

### 3.3. Scanning Electron Microscopy (SEM)

The composite films were fractured in liquid nitrogen to observe the interior of the unstressed composites. The fracture surfaces were observed by scanning electron microscopy (SEM) under high vacuum with a SEM instrument (S-3400N, Hitachi Ltd., Tokyo, Japan). Before the SEM observations, the fractured sample surfaces were sputter-coated with a 20 nm thick conductive gold layer.

### 3.4. Differential Scanning Calorimetry (DSC)

DSC analysis of PLA/PTMC/OEO composites was carried out in a DSC Q2000 device (TA Instruments, New Castle, DE, USA) under an inert nitrogen atmosphere flow rate of 100 mL/min. About 10 mg of specimen was heated from 20 °C to 220 °C at 10 °C/min to identify possible changes in crystallization and melting transitions due to aging. Subsequently, the sample was cooled to room temperature at a cooling rate of 10 °C/min and further heated to 220 °C at 10 °C/min. Peak temperature and peak areas were determined using the instrument software and the percentage of crystallinity (*X_c_*)) was calculated according to the following equation [44]:

*X_c_* (%) = (△H_m_/W△H^o^_m_) × 100 (1)

where △H_m_ (J/g) is the heat of fusion of the sample. W is the PLA weight fraction in the sample, and △H°_m_ is the theoretical heat of fusion of 100% crystalline PLA (93.7 J/g) [19].

### 3.5. Thermogravimetric Analysis (TGA)

Thermogravimetric analysis tests were carried out on a TGA Q5000 apparatus (TA Instruments). Approximately 10 mg samples were sealed in aluminum pans and heated from 20 to 600 °C at a heating rate of 10 °C/min in a nitrogen atmosphere. Weight losses of samples were measured as a function of temperature.

### 3.6. Color

The surface color of composite films was evaluated by measuring L* (light/dark), a* (red/green), and b* (yellow/blue) using a colorimeter (WSC-S; Shanghai Precision Instrument Co., Ltd., Shanghai, China). All measurements were performed in triplicate. Total color difference (Δ*E*) induced by oregano essential oil was calculated by the following equation:

Δ*E* = [(*L*_1_* − *L*_0_*)^2^ + (*a*_1_* − *a*_0_*)^2^ + (*b*_1_* − *b*_0_*)^2^]^1/2^ (2)

where *L*_0_*, *a*_0_*, and *b*_0_* was the color values of PLA/PTMC, and *L*_1_*, *a*_1_*, and *b*_1_* represented the color values of composite films of different amount oregano essential oil (3, 6, 9 and 12 wt %).

### 3.7. Antioxidant Activiyt

The radical scavenging activity of composite films was determined by a DPPH (2,2-diphenyl-picrylhydrzyl) free radical quenching assay, according to the method by Byun *et al*. [45], but with slight modifications. Approximately 100 mg of film samples were cut into small pieces and mixed with 2 mL methanol for 3 min. The mixture was left to stand for 3 h, at room temperature and then vigorously vortexed for 3 min. Supernatant liquor (0.5 mL) of the mixture solution was mixed with 2 mL of 0.06 mM DPPH in methanol. The mixture was vigorously stirred and kept in the dark at room temperature for 30 min. Absorbance was measured at 517 nm using a UV-visible spectrophotometer. All experiments were carried out in triplicate and the antioxidant capacity of film was expressed in % DPPH radical scavenging activity/2 mg of film. DPPH radical scavenging activity was calculated using the following equation:

Scavenging activity (%) = [(Abs_balnk_ − Abs_sample_)/Abs_balnk_] × 100 (3)

where Abs_blank_ is the absorbance of control and Abs_sample_ is the absorbance of the sample.

### 3.8. Determination of Total Phenolic (TP)

Quantification of the total soluble phenolic was estimated according to the Folin-Ciocalteu method. One hundred mg of each film sample was dissolved in 10 mL of distilled water for 24 h, and then the extract solution of the composite films (0.5 mL), 10% Folin-Ciocalteu reagent (2.5 mL), and 7.5% Na_2_CO_3_ solution (2 mL) were mixed at room temperature for 60 min. The absorbance values were then measured at 765 nm in a spectrophotometer (UV-VIS 1601, Shimadzu, Japan). A calibration curve was plotted using gallic acid at specific concentrations and the total phenolic content of the films was expressed as mg gallic acid equivalents (GAE) per gram of dried film according to the following equation:

T = C × V/M (4)

where T is total content of phenolics compound (milligramme per gram dried film, in GAE), C is the concentration of gallic acid obtained from the calibration curve (milligramme per milliliter), V is the volume of film extract (milliliter) and M is the weight of dried film (gram).

### 3.9. Antimicrobial Activity

The liquid culture test was used to evaluate the antimicrobial activity of the oregano essential oil in inhibiting the growth of two test bacterial microorganisms, *E. coli* and *L. monocytogenes*. All bacterial strains were obtained from the Laboratory of Microbiology, Kunming University of Science and Technology (Yunnan, China). The PLA/PTMC film was treated as the control group. The bacteria were activated by innoculation into Mueller-Hinton broth. A glass test tube containing test specimens (0.18–0.20 g for each) was filled with 10 mL of broth. The medium was inoculated with 0.1 mL of an overnight culture of bacteria. The bacterial cultures were adjusted to a cell concentration of 10^5^ CFU/mL [9]. Petri dishes were then incubated at 37 °C for 12 h. The antimicrobial activity of each sample was measured by examining the bacterial growth and colony-forming units (CFU) were counted.

### 3.10. Mechanical Properties

Mechanical properties of films, including tensile modulus, tensile strength (TS), and elongation at break (E), were measured using a universal tensile machine (CMT 4104, MTS Systems Co., Ltd., Shanghai, China). The measurements were conducted at a crosshead speed of 50 mm/min at room temperature according to ASTM D638. An average of six test values was taken for each sample.

### 3.11. Water Vapor Permeability (WVP)

Water vapor permeability (WVP) was determined gravimetrically using a water vapor transmission measuring cup in accordance with the ASTM E96-95 standard method [46]. Films were fastened on the top of the measuring cups which contain the desiccants. The covered acrylic cups were placed in a relative humidity chamber at 18 °C and relative humidity (RH) of 50%. The weight loss of the cup was considered equal with the transferred water through the film and adsorbed by desiccants. Weight loss of the measuring cup was measured as a function of time for 12 h. The WVP of the film was calculated as follows [47]:

*WVP* = (*WVTR* × *d*)/Δ*P* (5)

where *WVTR* is the water vapor transmission rate (g/m^2^·s), *d* is the film thickness (m), and Δ*P* is the partial pressure difference across the film (Pa).

### 3.12. Statistical Analysis

Data were analyzed by analysis of variance (ANOVA) using a statistical computer software package (SPSS version 13.0, IBM Co., Ltd, Chicago, IL, USA). The significance of differences between mean values was assessed using the Duncan’s multiple range test at a significance level of *p* < 0.05.

## 4. Conclusions

Composite films were prepared with PLA, PTMC and oregano essential oil by a solvent casting method. With the addition of OEO, the crystallinity of PLA phase decreased, but the flexibility and extensibility increased, the film cross-section became rougher, and the TP content and the DPPH scavenging activity of the films significantly increased (*p* < 0.05). However, the water barrier and optical properties did not change with varying contents of OEO. PLA/PTMC/OEO composite films containing 9 wt % exhibited strong antioxidant and significant antimicrobial effects against *E. coli* and *L. monocytogenes*. Therefore, the PLA/PTMC/OEO composite films might be commercially useful for active packaging applications. Additional studies are needed to evaluate the diffusion and release kinetics for the active release of the EO from the polymer matrix during food storage.

## Figures and Tables

**Table 1:** The thermal properties of PLA/PTMC/OEO composite films.

Sample	*T_g_* (°C)	*T_c_* (°C)	*T_m_* (°C)	*X_c_* (%)	*T_oneset_* (°C)
PLA/PTMC	46.7	105.4	167.2	20.68	293.1
PLA/PTMC/O3	54.4	110.1	167.5	19.78	284.2
PLA/PTMC/O6	51.4	104.6	164.5	19.03	280.9
PLA/PTMC/O9	49.6	100.3	161.8	19.80	278.3
PLA/PTMC/O12	45.6	84.2	156.5	11.79	270.9

**Table 2:** The visual properties of PLA/PTMC/OEO composite films.

Sample	*L*	*a*	*b*	*E*
PLA/PTMC	73.65 ± 0.51 ^a,b^	1.26 ± 0.08 ^a^	−0.15 ± 0.08 ^a^	-
PLA/PTMC/O3	73.36 ± 0.71 ^a^	1.18 ± 0.13 ^a,b^	−0.14 ± 0.12 ^a^	0.31
PLA/PTMC/O6	74.47 ± 0.27 ^b,c^	1.38 ± 0.21 ^a^	−0.2 ± 0.06 ^a^	0.83
PLA/PTMC/O9	74.69 ± 0.18 ^c^	1.33 ± 0.17 ^a^	0.21 ± 0.23 ^b^	1.10
PLA/PTMC/O12	73.30 ± 0.47 ^a^	0.73 ± 0.46 ^b^	−0.14 ± 0.09 ^a^	0.63

^a−c^ Values followed by different letters in the same column were significantly different (*p* < 0.05), where ^a^ is the lowest value.

**Table 3:** The mechanical properties of PLA/PTMC/OEO composite films.

Sample	Tensile Strength (MPa)	Elongation at Break (%)	Modulus of Elasticity (MPa)
PLA/PTMC	12.38 ± 1.15 ^a^	105.51 ± 29.03 ^a^	373.78 ± 57.67 ^a^
PLA/PTMC/O3	13.00 ± 1.32 ^a^	177.99 ± 24.17 ^b^	539.25 ± 89.45 ^b^
PLA/PTMC/O6	11.93 ± 0.94 ^a,b^	181.43 ± 32.53 ^c^	363.37 ± 43.43 ^a^
PLA/PTMC/O9	10.07 ± 0.95 ^b^	186.70 ± 29.03 ^c^	266.55 ± 69.33 ^a^
PLA/PTMC/O12	11.29 ± 1.56 ^a,b^	190.84 ± 13.37 ^d^	329.98 ± 78.81 ^a^

^a,b^ Values followed by different letters in the same column were significantly different (*p* < 0.05), where ^a^ is the lowest value.
